# Investigation and characterization of changes in potato peels by thermochemical acidic pre-treatment for extraction of various compounds

**DOI:** 10.1038/s41598-024-63364-6

**Published:** 2024-06-02

**Authors:** Qudsia Mushtaq, Uzair Ishtiaq, Nicolas Joly, Patrick Martin, JavedIqbal Qazi

**Affiliations:** 1 Institute of Zoology, Microbial Biotechnolog Laboratory, University of the Punjab, Lahore, 54590 Pakistan; 2 Department of Research and Development, Paktex Industries, 2.5 KM Tatlay Road, Kamoke, 52470 Gujranwala Pakistan; 3 Unite Transformations & Agroresources - ULR7519, Univ. Artois, UniLaSalle, 62408 Bethune, France; 4 Department of Life Sciences, University of Management and Technology, Lahore, Pakistan

**Keywords:** BBD, Fermentable sugars, SEM, Sulphuric acid (H_2_SO_4_), RSM, Plant base-phenolic compounds, FTIR, Potato Peel, Biological techniques, Biotechnology, Chemical biology, Plant sciences

## Abstract

Potato peel waste (PPW) is an underutilized substrate which is produced in huge amounts by food processing industries. Using PPW a feedstock for production of useful compounds can overcome the problem of waste management as well as cost-effective. In present study, potential of PPW was investigated using chemical and thermochemical treatment processes. Three independent variables i.e., PPW concentration, dilute sulphuric acid concentration and liberation time were selected to optimize the production of fermentable sugars (TS and RS) and phenolic compounds (TP). These three process variables were selected in the range of 5–15 g w/v substrate, 0.8–1.2 v/v acid conc. and 4–6 h. Whole treatment process was optimized by using box-behnken design (BBD) of response surface methodology (RSM). Highest yield of total and reducing sugars and total phenolic compounds obtained after chemical treatment was 188.00, 144.42 and 43.68 mg/gds, respectively. The maximum yield of fermentable sugars attained by acid plus steam treatment were 720.00 and 660.62 mg/gds of TS and RS, respectively w.r.t 5% substrate conc. in 0.8% acid with residence time of 6 h. Results recorded that acid assisted autoclaved treatment could be an effective process for PPW deconstruction. Characterization of substrate before and after treatment was checked by SEM and FTIR. Spectras and micrographs confirmed the topographical variations in treated substrate. The present study was aimed to utilize biowaste and to determine cost-effective conditions for degradation of PWW into value added compounds.

## Introduction

Indecent dumping of wastes in many urban areas results into contaminated environment raising health concerns. Incineration and landfilling are generally employed practices for solid waste management in Pakistan^1,2^. Microbial spoilage of dumped food waste creates severe environmental concern. Industrial application of food wastes appears appealing to provide cost effective substrates as well as to solve the environmental issue. PPW can be converted into various useful products like enzymes, antioxidants, reducing sugars, biogas, biofuel, bio-fertilizers and bioabsorbent^3–6^.

Approximately, one-third of global food produced is estimated to be lost^7^ and these wastes have been cost at 1 trillion USD^8^.

Potato is a main edible crop with annual production of 370 million tons^9^. A major part of potato is used as fresh product whereas the residual part is used and consumed as a dried potatoes, starch, crisp, and fries. During processing of potato, 15 to 40% of the initial weight is removed from fresh potatoes and is termed as PPW^10,11^. Food industry especially the fast food restaurants produce heavy amounts of potato peels. Collection of PPW from such units within a cosmopolitan city would suffice to provide raw material for a second generation bioethanol production unit.

Phenolic compounds are abundantly present in plants and their antioxidant properties prevent the cell from oxidative damage and eventually lessen the burden of chronic and cardiovascular diseases. Synthetic antioxidants abundantly being used in pharmaceutical and food industries to preserve drug and food products and reported to induce various carcinomas. Consequently, the natural antioxidants are being prepared^12^. In this paper we explored the potential of potato skins as a precursor of various bioactive ingredients which have diverse biotechnological usage. The practical approach is to develop the awareness of proper and sustainable management of agricultural waste as well as the synthesis of various compounds such as lactic acid, biohydrogen, bioabsorbent, enzymes and various food additives which will serve as an excellent source to develop link between different industries.

Various research reports have reported the production of useful compounds from PPW and their use in various sectors, particularly food processing industry such as formulation of yogurt, functional biscuits, fermented juice etc. Addition of potato peel extract in different food products has enhanced the vitamin content, shelf life, antioxidant profile, color and sensory properties^13–17^. The main purpose of this work was to produce fermentable sugars which can enhance biofuel production from PPW. For microbial fermentation small monomeric sugars are more accessible as compared to complex polymers. PPW conversion into biofuels and biogas offers exceptional alternatives to fossil fuels. Previously, many research reports have confirmed this conversion and have recorded good yield of biofuel employing various lucrative substrates^5,18–20^.

Few novel sophisticated processes for example acidified solvents based extraction^21^, microwave assisted and ultrasonic extractions^22,23^ and liquid based pressurized removal^24^ have been introduced to extract the valuable compounds from PPW. But these methods are highly expensive and complex and the present research has reported time saving, low cost and very simple methods to isolate useful compounds from PPW. Different strategies have been applied for process optimization using ANN (artificial neural network), RSM (response surface methodology), GA (genetic algorithm) and OFAT (one factor at a time)^25–27^. OFAT approach is a time consuming method which often leads to inaccurate results, whereas RSM is an efficient statistical model that analyses the individual and combined effect of different parameters on the yield^28^. Therefore, scientists are currently using RSM to enhance product yield^29,30^. Acid and steam assisted acid treatments to obtain value-added products employing various plant biowaste are cost effective approaches in select situations to obtain some useful products^31–33^.

This work was aimed to optimize acidic treatment by using box-behnken design to optimize yield of TS, TP and RS. Of different food wastes, potato peels represent starch rich, antioxidant as well as phenolic compounds and dietary fibers harboring cost-effective resource^34–36^. The process developed in this work is economical for the conversion of a food waste into useful products such as phenolic compounds and fermentable sugars. This effort is promising to provide cheaper feedstock for various biotechnological applications particularly second generation biofuel. Accessory fermentation residues enriched in single cell protein can be used as an excellent ingredient in animal feed.

## Materials and methods

### Potato peel drying

PPW were obtained from local fries shop (Gate No.4, Quaid-e-azam campus University of Punjab, Lahore, Pakistan. They were properly washed to remove all dirt, sun dried for few days and then oven dried at 60 °C for 24 h till consistent weight. The processed PPW were powdered by electric mill and sieved by strainer to obtain the uniform size of 1–2 mm. The powdered PPW was stored in air tightened containers at room temperature (25 °C ± 5 °C).

### Proximate composition of dried potato peels

The compositional analysis of PPW was characterized by determining the content of different components including moisture, ash, crude fat, crude protein, crude fiber and carbohydrates using standard protocols of AOAC^37^.

### Pretreatment

**Figure 1:**
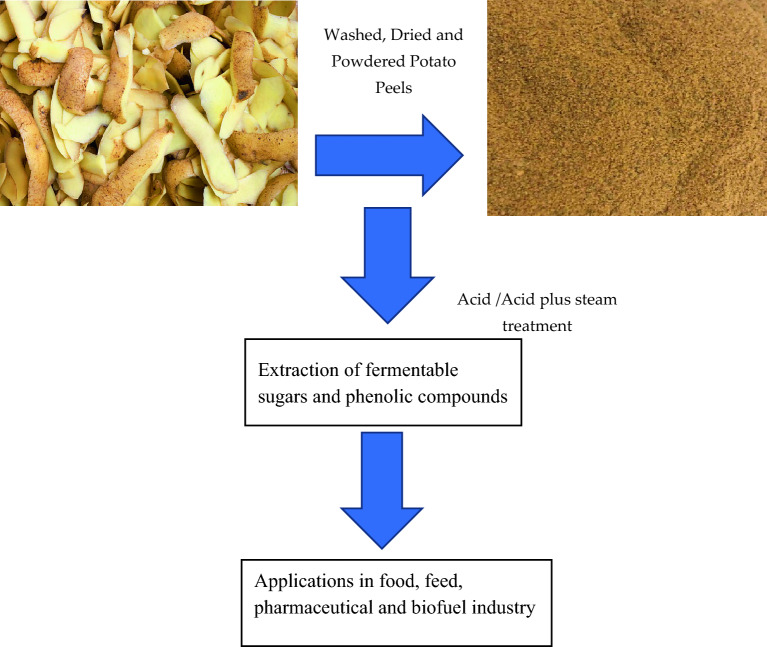
Low cost production of valuable compounds from PPW.

Erlenmeyer flasks were used as pretreatment reactors with a working volume up to 100 ml. Different concentrations of substrate were soaked into different concentration of acid for various time periods, and then autoclaved at 121 °C, 21 psi for 15 min. After autoclaving, the samples were carefully filtered and washed up to neutrality. The clear filtrate free of any solid residue was used to determine the value of sugars and phenolic compounds (Fig. 1).

### Experimental Design

To optimize the treatment, box-behnken design was used. Some independent variables including substrate conc. (K_1_), acid conc. (K_2_) and the time (K_3_) were selected in the range of 5–15 g, 0.8–1.2% and 4–8 h, respectively as described in the Table 1.

**Table 1:** Range of the parameters used for BBD.

Parameter	Code	Level		
		− 1	0	1
Substrate conc. (%)	K_1_	5	10	15
H2SO4 conc. (%)	K_2_	0.8	1	1.2
Time (hour)	K_3_	4	6	8

The yield of dependent variable was calculated by using Statistical Software Minitab (2016), Version 16. Minitab Incorporation, State College from the following equation.

1 $${\text{I}} = {\text{J}}_{0} + {\text{J}}_{{1}} {\text{K}}_{{1}} + {\text{J}}_{{2}} {\text{K}}_{{2}} + {\text{J}}_{{3}} {\text{K}}_{{3}} + {\text{J}}_{{{11}}} {\text{K}}_{{{12}}} + {\text{J}}_{{{22}}} {\text{K}}_{{{22}}} + {\text{J}}_{{{33}}} {\text{K}}_{{{32}}} + {\text{J}}_{{{12}}} {\text{K}}_{{1}} {\text{K}}_{{2}} + {\text{J}}_{{{12}}} {\text{K}}_{{1}} {\text{K}}_{{2}} + {\text{J}}_{{{13}}} {\text{K}}_{{1}} {\text{K}}_{{3}} + {\text{J}}_{{{23}}} {\text{K}}_{{2}} {\text{K}}_{{3}}$$

Here response is the I, J_0_ is constant, linear coefficients are J_1_K_1_ to J_3_K_3_, quadratic coefficients are J_11_K_12_ to J_33_K_32_, interactive coefficients are J_12_K_1_K_2_ to J_23_K_2_K_3_.

### Substrate characterization

Raw and acid treated samples with maximum fermentable sugars and phenolic content from both chemical and thermochemical treatment were further characterized through fourier transform infrared spectroscopy (FTIR Agilent Technologies Cary630, Santa Clara, USA) to determine the vibration frequency in functional groups. The spectra were measured with a resolution of 4 cm^−1^ and in the range 3500–500 cm^−1^. The samples were ground into fine powder using an electric miller and subjected to analysis.

Scanning electron microscopy (SEM JSM-6490LA, Japan) was carried out to confirm the visual deformation to the ultrastructure of PPW. Samples were mounted on aluminum stubs with double-sided carbon adhesive tabs. Specimens were observed using Jeol JSM-6490LA scanning microscope (Tokyo,Japan) at an accelerating voltage of 1.7 kV.

### Analytical methods

After completion of pretreatment process, total and reducing sugars and phenolic compounds were measured. Reducing sugar and total sugar were determined by DNS using Miller^38^ and Dubois method^39^, respectively. For the determination of phenolic compounds, Carralero Sanz’s method^40^ method was used.

### Acid saccharification of PPW

After acid hydrolysis, saccharification of substrate was determined at various time periods such as 4,6,8 and 10 h. The 250 ml Erlenmeyer flasks were used to conduct experiment containing of acid treated and acid assisted autoclaved treated PPW (5 g). For determination of reducing sugar samples were regularly taken. Saccharification (%) was calculated by using below formula^41^.

$$saccharification (\%)=\frac{Reducing\, sugars}{Substrate\, used}$$

### Mass balance

Once pretreatment process is completed, liquid that was obtained was filtered with the help of Whatman filter paper No. 1 to measure mass balance. Residue left on filter paper was washed with 1 M NaOH to neutralize the pH. Afterwards, filter paper having treated substrate were placed at 50 °C in oven until consistent weight was attained. The solubility index (S.I) was measured by following formula:

$$\left({\text{S.I.}}\right)=\frac{{\text{Initial.\, Weight}}.-{\text{Final.\, Weight}.}}{{\text{Final.\, Weight}}.}\times 100$$

### Ethical concern

There was no use of animal or human in this study, so no ethical concerns was raised during this study.

### Research involving plants

The potato peels used of in this study complied with national and international guidelines.

## Results and discussion

During this work, pulverized PPW were pretreated by dilute sulphuric acid and acid plus steam. The experiments were conducted in two batches. In first batch of chemical treatment the substrate was treated with dilute acid. In the second batch, thermochemical treatment was applied. Diltue Sulphuric acid treatment was followed by autoclaving at 121 °C, at 15psi for 15 min. The main objective of this experiment was to get maximum degradation of potato peels into RS, TS and TP.

### Proximate composition of dried PPW

The chemical composition of potato peels was characterized by proximate analysis expressed as a percentage of weight is shown in Table 1. Proximate analysis reported the incidence of various useful compounds, it comprised moisture content up to 10.29%, ash quantity 8.81%, crude fat 0.44%, crude protein 15.45%, crude fiber 4.4% and carbohydrates 65.01% on dry weight basis.

Carbohydrates were found to be the main component accounting for 65% of the potato peels. Among the carbohydrates, the main component was starch. A work by Kpanja et al.^42^ has reported approximately similar value of ash content i.e., 7.11% while determining the proximate composition of sun-dried irish potat peels. The quantity of carbohydrate which is mainly starch was in accordance with the value reported by Badr SA and El-Wasif^43^. Another research report by Adegunloye and Oparinde 2017^44^ has recorded the carbohydrate content of 72.60% in potato peels while determining the effects of fermentation on the proximate composition of Irish (*Solanum tuberosum*) peels.

This report measured the protein content of 15.45% of potato peels. A research report by Maxwell et al.^45^ has measured the protein content of 12.5% while reporting the effect of Saccharomyces cerevisiae on protein enrichment of potato peels using solid-state fermentation process. Onuguh et al.^46^ have recorded almost similar level of fat from potato peels that is 0.28%. Moisture, fiber and ash values were almost agreed with values reported by Rowayshed et al.^47^.

The notably higher quantity of carbohydrate rendered the waste biomass a lucrative medium encompassing the key nutrients essential for production of valuable compounds. In addition, this waste also contained rich amount of starch, which could serve as an efficient feedstock for biofuel production (Table 2).

**Table 2:** Physico-chemical characterization of potato peel.

Sr no	Parameters (%)	PPW
1	Moisture	10.29 ± 0.12
2	Ash	8.81 ± 0.09
3	Crude fat	0.44 ± 0.03
4	Crude protein	15.45 ± 0.21
5	Crude fiber	4.4 ± 0.08
6	Carbohydrates	65.01 ± 0.19

Values are mean of triplicates values ± standard deviation.

To optimize the pretreatment conditions, BBD with three values and three factors was applied. The independent factors such as substrate concentration (K_1_), sulphuric acid loading (K_2_) and (K_3_) as the time were selected for the pre-treatment. These factors with ranges and codes are mentioned in the Table 1. After each pre-treatment, reducing and total sugars and phenolic compounds were calculated. The extraction yield was expressed in mg/gds (gram dry substrate). Result of these analyses is described in Tables 3 and 4. The response was calculated by second order polynominal equation (Eq. 2–7).

**Table 3:** BBD on extraction yields of fermentable sugars and phenolic compounds after acid treatment of PPW.

Run No.	K_1_	K_2_	K_3_	Total Sugar mg/gds			Reducing Sugar mg/gds			Total Phenolic Compounds (mg/gds)		
				Predict	Observe	Difference	Predict	Observe	Difference	Predict	Observe	Difference
1	5	0.8	6	95.6	84.00	11.6	31.40	26.10	−5.3	35.63	40.54	4.906
2	5	1.2	6	168.16	188.00	19.84	61.14	58.48	−2.66	38.76	43.68	4.92
3	15	0.8	6	147.74	142.93	−4.81	30.53	31.41	0.883	15.8	14.16	−1.64
4	15	1.2	6	66.833	72.733	5.9	34.173	35.95	1.777	14.10	12.46	−1.635
5	10	0.8	4	162.16	172.16	10	41.20	43.94	2.74	17.86	17.36	−0.506
6	10	1.2	4	39.41	29.41	−10	50.44	51.86	1.42	17.11	16.60	−0.513
7	10	0.8	8	64.98	74.98	10	78.80	77.37	−1.43	17.78	18.30	0.514
8	10	1.2	8	102.63	98.10	−4.53	95.21	92.46	−2.75	17.55	18.06	0.507
9	5	1.0	4	129.4	121.2	−8.2	56.80	56.6	−0.2	24.09	20.2	−3.892
10	15	1.0	4	95.013	97.866	2.853	45.66	42.95	−2.713	11.88	13.86	1.978
11	5	1.0	8	61.15	62.9	1.75	136.10	144.12	8.02	28.37	22.44	−5.932
12	15	1.0	8	95.13	97.866	2.736	60.10	63.12	3.024	10.70	12.00	1.298
13	10	1.0	6	124.63	174.10	49.47	52.30	43.68	−8.62	14.31	12.60	−1.71
14	10	1.0	6	124.63	89.30	−35.33	38.25	43.68	5.43	14.31	13.90	−0.41
15	10	1.0	6	124.63	160.50	35.87	40.50	43.68	3.18	14.31	16.43	2.12

**Table 4:** BBD on extraction yields of fermentable sugars and phenolic compounds after acid plus steam treatment of PPW.

Sample No.	K_1_	K_2_	K_3_	Total Sugar mg/gds			Reducing Sugar mg/gds			Total Phenolic Comopunds mg/gds		
				Predict	Observe	Difference	Predict	Observe	Difference	Predict	Observe	Difference
1	5	0.8	6	791.04	720.00	−71.04	699.00	660.62	−38.38	39.90	38.66	−1.24
2	5	1.2	6	619.66	584.0	−35.66	612.26	513.4	−98.86	37.02	37.46	0.44
3	15	0.8	6	467.46	474.68	7.22	358.41	391.37	32.96	19.00	18.86	−0.14
4	15	1.2	6	469.93	493.61	23.68	321.40	334.2	12.8	25.113	25.53	0.417
5	10	0.8	4	435.99	457.01	21.02	392.22	357.04	−35.18	21.79	22.17	0.38
6	10	1.2	4	351.20	354.51	3.31	346.96	342.02	−4.94	27.37	26.91	−0.46
7	10	0.8	8	281.92	278.62	−3.3	248.85	253.80	4.95	25.14	25.61	0.47
8	10	1.2	8	284.72	263.71	−21.01	195.21	230.40	35.19	27.28	26.90	−0.38
9	5	1.0	4	549.82	578.84	29.02	345.26	454.02	108.76	34.1	34.6	0.5
10	15	1.0	4	391.02	365.14	−25.88	312.76	303.26	−9.5	20.64	20.53	−0.11
11	5	1.0	8	251.76	329.42	77.66	279.10	307.62	28.52	37.46	37.80	0.34
12	15	1.0	8	343.34	333.67	−9.67	138.07	101.82	−36.25	21.69	21.53	−0.16
13	10	1.0	6	271.42	238.91	−32.51	209.87	210.31	0.44	26.40	29.12	2.72
14	10	1.0	6	271.42	248.73	−22.69	209.87	184.80	−25.07	26.40	24.80	−1.6
15	10	1.0	6	271.42	366.62	95.2	209.87	234.51	24.64	26.40	25.30	−1.1

### Chemical and thermochemical pretreatment effects on the yield

It is clearly demonstrated that the chemical treatments produce maximum production of total sugar upto 188 mg/gds corresponding to 5% w/v of substrate loading in 1.2% v/v of sulphuric acid conc. for 6 h. Optimum production of reducing sugars up to 144.12 mg/gds and phenolic compounds up to 43.68 mg/gds were liberated at run No. 11 and 2, respectively. Thermochemical (acid assisted steam) treatment as described in Table 4 has yielded high production of fermentable sugars and phenolic compounds. It has reported 720.00 mg/gds of total sugars using 5% w/v of substrate loading in 0.8% v/v of sulphuric acid for 6 h. Optimum extraction of reducing sugars up to 660.62 mg/gds and phenolic compounds up to 38.66 mg/gds were observed at run No. 1. It was proved that use of dilute acid assisted autoclaved treatment was more useful in monomerization pf polymeric sugars of PPW. Alot of research was reported that have high record of production of reducing sugar and polysaccharides from dilute acid and dilute acid plus heat-treated PPW for the production of biofuels^48–51^.

Significant response was indicated by regression analysis. Squared, interactive and linear effetct of process parameters was measured using analyses of variance (ANOVA). During treatment T.S, R.S and T.P were found significant with p-values of 0.004, 0.043 and 0.056, respectively as depicted in Table 5. During acid treatment the determination of coefficient (R_2_) values of 89.71%, 97.62% and 76.11% were found significant for T.S, R.S and T.P, respectively predicted the accuracy of the model.

**Table 5:** Analysis of the variance after chemical treatment.

T.S. (mg/gds)	Origin	D.F	Adj. M.S	Adj. S.S	P. values	F. values
	Regression	9	453.318	4079.86	0.002	18.71
	Linear	3	299.649	898.95	0.009	12.37
	K_1_ (%)	1	312.716	312.72	0.016	12.90
	K_2_ (%)	1	639.374	639.37	0.004	26.38
	K_3_ (h)	1	2.095	2.09	0.781	0.09
	Square	3	431.200	1293.60	0.004	17.79
	K_1_ * K_1_ (%)	1	444.022	444.02	0.008	18.32
	K_2_ * K_2_ (%)	1	795.552	795.55	0.002	32.83
	K_3_ * K_3_ (h)	1	67.066	67.07	0.157	2.77
	Interaction	3	18.059	54.18	0.570	0.75
	K_1_ * K_2_	1	19.976	19.98	0.406	0.82
	K_1_ * K_3_	1	19.180	19.18	0.414	0.79
	K_2_ * K_3_	1	15.020	15.02	0.467	0.62
	Error	5	24.234	121.17		
	Lack-of-fit	3	24.993	74.98		1.08
	Pure Error	2	23.094	46.19	0.002	
	Total	14				

R.S. (mg/gds)	Origin	D.F.	Adj. M.S	Adj. S.S.	P values	F values
	Regression	0	204.628	1841.65	0.043	5.13
	Linear	3	166.375	499.12	0.079	4.17
	K_1_ (%)	1	135.216	135.22	0.125	3.39
	K_2_ (%)	1	61.422	61.42	0.270	1.54
	K_3_ (h)	1	220.122	220.12	0.066	5.52
	Square	3	234.335	703.01	0.043	5.88
	K_1_ * K_1_ (%)	1	398.091	398.09	0.025	9.99
	K_2_ * K_2_ (%)	1	208.438	208.44	0.071	5.23
	K_3_ * K_3_ (h)	1	84.692	84.69	0.205	2.12
	Interaction	3	79.728	239.19	0.233	2.00
	K_1_ * K_2_	1	0.369	0.37	0.927	0.01
	K_1_ * K_3_	1	0.176	0.18	0.950	0.00
	K_2_ * K_3_	1	238.641	238.64	0.058	5.99
	Error	5	39.868	199.34		
	Lack-of-fit	3	61.167	183.50	0.117	7.72
	Pure Error	2	7.919	15.84	0.043	5.13
	Total	14				

T.P. (mg/gds)	Origin	D.F.	Adj. M.S.	Adj. S.S.	P values	F values
	Regression	9	0.506793	4.56114	0.002	19.59
	Linear	3	0.050603	0.15181	0.239	1.96
	K_1_ (%)	1	0.068121	0.06812	0.166	2.63
	K_2_ (%)	1	0.010953	0.01095	0.544	0.42
	K_3_ (h)	1	0.055099	0.05510	0.204	2.13
	Square	3	0.019187	0.05756	0.571	0.74
	K_1_ * K_1_ (%)	1	0.001010	0.00101	0.851	0.04
	K_2_ * K_2_ (%)	1	0.006119	0.00612	0.647	0.24
	K_3_ * K_3_ (h)	1	0.050941	0.05094	0.220	1.97
	Interaction	3	0.103560	0.31068	0.085	4.00
	K_1_ * K_2_	1	0.280900	0.28090	0.022	10.86
	K_1_ * K_3_	1	0.029756	0.02976	0.333	1.15
	K_2_ * K_3_	1	0.000025	0.00002	0.976	0.00
	Error	5	0.025875	0.12938		
	Lack-of-fit	3	0.005898	0.01769	0.949	0.11
	Pure Error	2	0.055841	0.11168		
	Total	14				

For acid plus steam treatment this model was found significant for the extraction of T.S, R.S, and T.P.C. The value of *Pred.* R^2^ and *Adj.* R^2^ were found as were found as 0.82, 0.59 and 0.68 and 0.93, 0.71 and 0.00 for R.S., T.S., and T.P.C, respectively.

**Figure 2:**
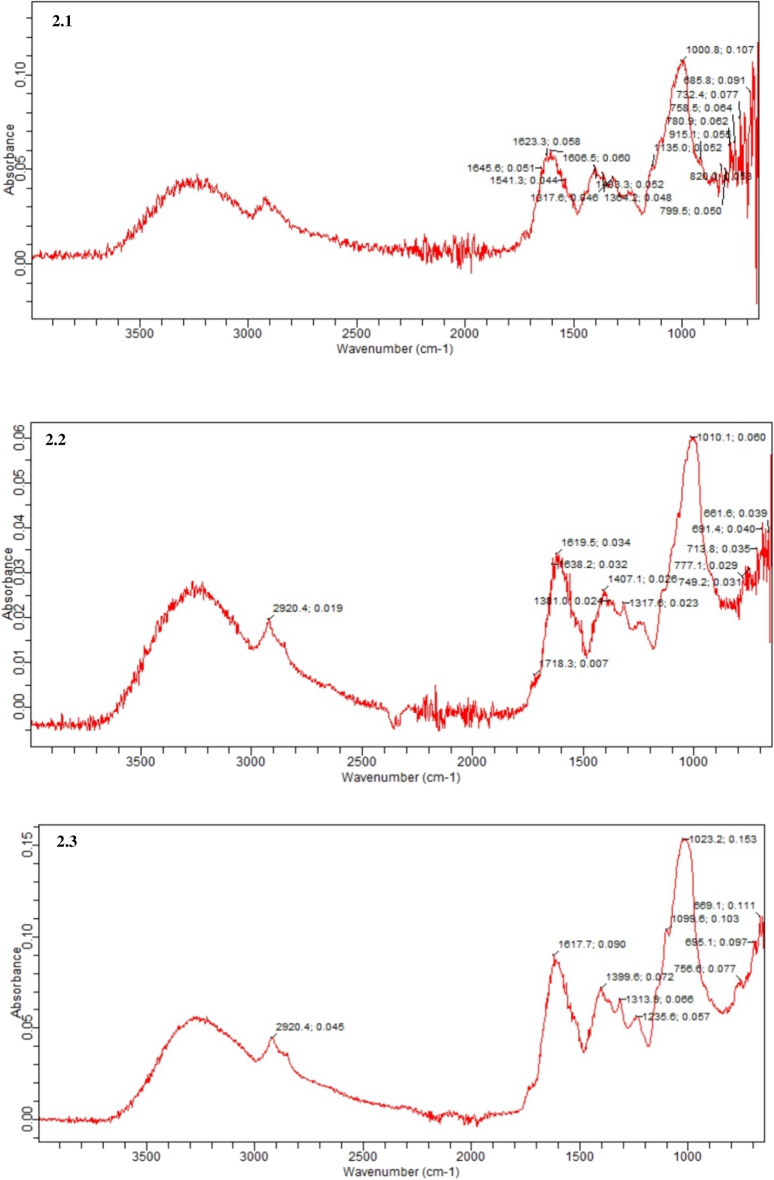
FTIR absorption spectra; (**a**) Control, (**b**) Acid treated, and (**c**) Acid assisted autoclaved PPW.

**Figure 3:**
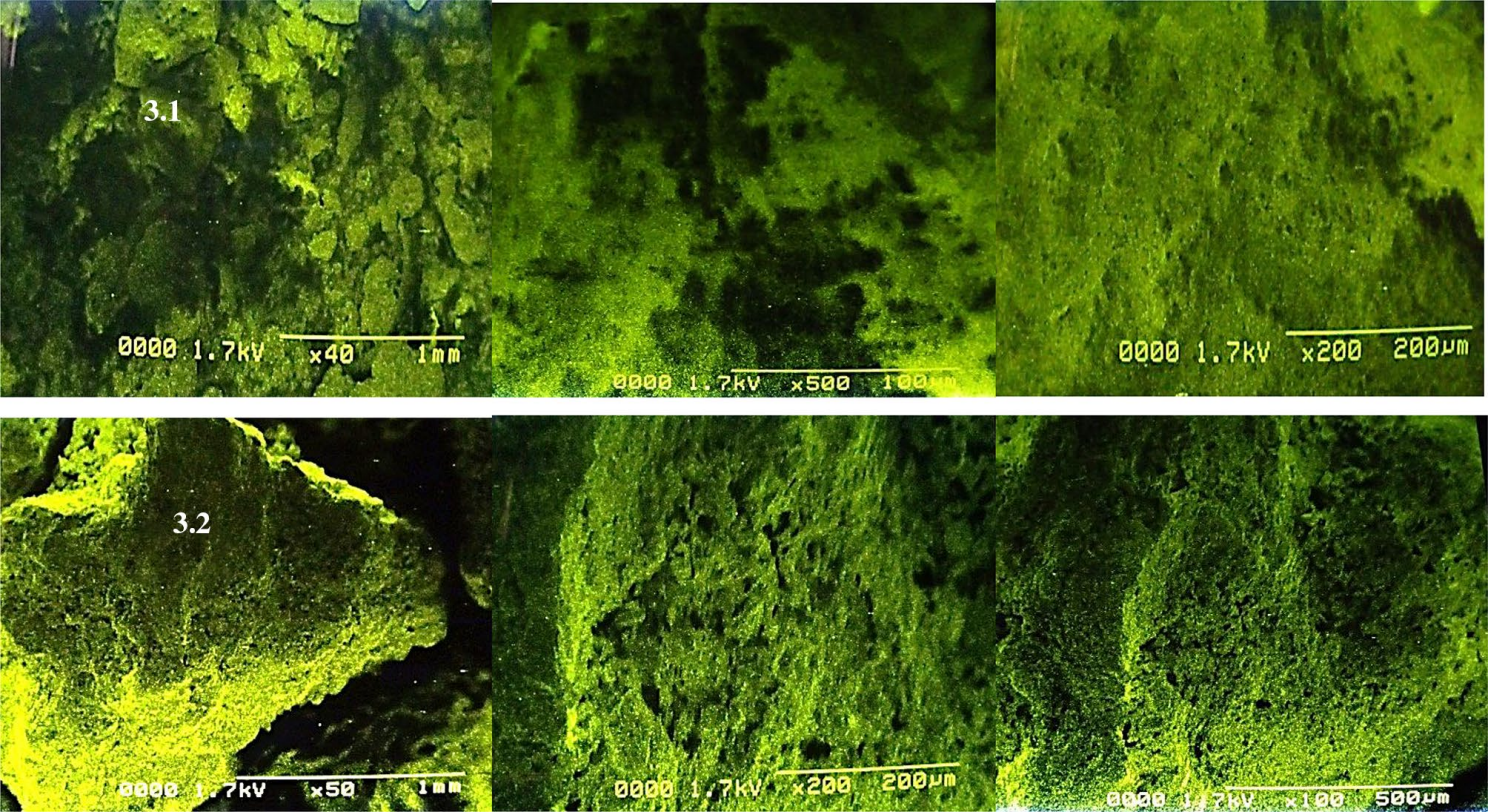
Scanning electron micrographs of control (**a**) and acid assisted autoclaved treated PPW (**b**).

In thermo-chemical methods, values of T.S, R.S, and T.P.C were observed as 32.83, 9.99 and 19.59, respectively by using Fisher’s f-test. While p-values of 0.004, 0.025, 0.022 were found significant for T.S., R.S. and T.P., respectively as depicted in Table 6. The R_2_ values of 97.12%, 90.23% and 97.24% for T.S., R.S. and T.P., respectively established the precision of model in acid plus steam treatment. The value of *Pred.* R^2^ and *Adj.* R^2^ were found as were found as 0.58, 0.65 and 0.82 and 0.81, 0.88 and 0.92 for R.S., T.S., and T.P.C, respectively (Figs. 2, 3).

**Table 6:** Variance analysis after thermo-chemical treatment.

T.S. (mg/gds)	Origin	D.F	Adj. M.S	Adj. S.S	P. Value	F. Value
	Regression	9	453.318	4079.86	0.002	18.71
	Linear	3	299.649	898.95	0.009	12.37
	K_1_ (%)	1	312.716	312.72	0.016	12.90
	K_2_ (%)	1	639.374	639.37	0.004	26.38
	K_3_ (h)	1	2.095	2.09	0.781	0.09
	Square	3	431.200	1293.60	0.004	17.79
	K_1_ * K_1_ (%)	1	444.022	444.02	0.008	18.32
	K_2_ * K_2_ (%)	1	795.552	795.55	0.002	32.83
	K_3_ * K_3_ (h)	1	67.066	67.07	0.157	2.77
	Interaction	3	18.059	54.18	0.570	0.75
	K_1_ * K_2_	1	19.976	19.98	0.406	0.82
	K_1_ * K_3_	1	19.180	19.18	0.414	0.79
	K_2_ * K_3_	1	15.020	15.02	0.467	0.62
	Error	5	24.234	121.17		
	Lack-of-fit	3	24.993	74.98		1.08
	Pure Error	2	23.094	46.19		
	Total	14				

R.S. (mg/gdsl)	Origin	D.F.	Adj. M.S.	Adj. S.S.	P value	F value
	Regression	9	204.628	1841.65	0.043	5.13
	Linear	3	166.375	499.12	0.079	4.17
	K_1_ (%)	1	135.216	135.22	0.125	3.39
	K_2_ (%)	1	61.422	61.42	0.270	1.54
	K_3_ (h)	1	220.122	220.12	0.066	5.52
	Square	3	234.335	703.01	0.043	5.88
	K_1_ * K_1_ (%)	1	398.091	398.09	0.025	9.99
	K_2_ * K_2_ (%)	1	208.438	208.44	0.071	5.23
	K_3_ * K_3_ (h)	1	84.692	84.69	0.205	2.12
	Interaction	3	79.728	239.19	0.233	2.00
	K_1_ * K_2_	1	0.369	0,37	0.927	0.01
	K_1_ * K_3_	1	0.176	0.18	0.950	0.00
	K_2_ * K_3_	1	238.641	238.64	0.058	5.99
	Error	5	39.868	199.34		
	Lack-of-fit	3	61.167	183.50	0.117	7.72
	Pure Error	2	7.919	15.84		5.13
	Total	14				

T.P. (mg/gds)	Origin	D.F.	Adj. M.S.	Adj. S.S.	P value	F value
	Regression	9	0.506793	4.56114	0.002	19.59
	Linear	3	0.50603	0.15181	0.239	1.96
	K_1_ (%)	1	0.068121	0.06812	0.166	2.63
	K_2_ (%)	1	0.010953	0.01095	0.544	0.42
	K_3_ (h)	1	0.055099	0.05510	0.204	2.13
	Square	3	0.019187	0.05756	0.571	0.74
	K_1_ * K_1_ (%)	1	0.001010	0.00101	0.851	0.04
	K_2_ * K_2_ (%)	1	0.006119	0.00612	0.647	0.24
	K_3_ * K_3_ (h)	1	0.050941	0.05094	0.220	1.97
	Interaction	3	0.103560	0.31068	0.085	4.00
	K_1_ * K_2_	1	0.280900	0.28090	0.022	10.86
	K_1_ * K_3_	1	0.029756	0.02976	0.333	1.15
	K_2_ * K_3_	1	0.000025	0.00002	0.976	0.00
	Error	5	0.025875	0.12938		
	Lack-of-fit	3	0.005898	0.01769	0.949	0.11
	Pure Error	2	0.055841	0.11168		
	Total	14

### Regression equation of chemical treatment

The regression model for the production of T.S., R.S., and T.P. is expressed as follows:

2 $${\text{I}}\left( {{\text{T}}.{\text{S}}.\;{\text{mg}}/{\text{gds}}} \right) = {12}.{4633} - {2}.{1275}\;{\text{K}}_{{1}} + {4}.{7488}\;{\text{K}}_{{2}} - 0.{8487}\;{\text{K}}_{{3}} - 0.{7}00{4}\;{\text{K}}^{{{2}/}}_{{1}} - 0.{4179}\;{\text{K}}^{{{2}/}}_{{2}} - {2}.{5329}\;{\text{K}}^{{{2}/}}_{{3}} - {3}.{94}00{5}\;{\text{K}}_{{1}} {\text{K}}_{{2}} + {4}.0{1}00\;{\text{K}}_{{1}} {\text{K}}_{{3}} + 0.{8575}\;{\text{K}}_{{2}} {\text{K}}_{{3}}$$

3 $${\text{I}}\left( {{\text{R}}.{\text{S}}.\;{\text{mg}}/{\text{gds}}} \right) = {4}.{3683} - 0.{5751}\;{\text{K}}_{{1}} - {1}.{469}0\;{\text{K}}_{{2}} + {1}.{85}0{9}\;{\text{K}}_{{3}} - 0.{3153}\;{\text{K}}^{{{2}/}}_{{1}} - 0.{469}\;{\text{K}}^{{{2}/}}_{{2}} + {2}.{588}\;{\text{K}}^{{{2}/}}_{{3}} - 0.{2345}\;{\text{K}}_{{1}} {\text{K}}_{{2}} + 0.{1793}\;{\text{K}}_{{1}} {\text{K}}_{{3}} - 0.{3375}\;{\text{K}}_{{2}} {\text{K}}_{{3}}$$

4 $${\text{I}}\left( {{\text{T}}.{\text{P}}.\;{\text{mg}}/{\text{gds}}} \right) = {1}.{431}0 - 0.0{246}\;{\text{K}}_{{1}} + 0.{1913}\;{\text{K}}_{{2}} + 0.00{9}0\;{\text{K}}_{{3}} + 0.{4376}\;{\text{K}}^{{{2}/}}_{{1}} + 0.{1826}\;{\text{K}}^{{{2}/}}_{{2}} - 0.{11}0{6}\;{\text{K}}^{{{2}/}}_{{3}} {-}0.{1}0{27}\;{\text{K}}_{{1}} {\text{K}}_{{2}} + 0.0{13}0\;{\text{K}}_{{1}} {\text{K}}_{{3}} {-}0.0{98}0\;{\text{K}}_{{2}} {\text{K}}_{{3}}$$

### Regression equation of thermo-chemical treatments

5 $${\text{I}}\left( {{\text{T}}.{\text{S}}.\;{\text{mg}}/{\text{gds}}} \right) = {397}.{21} - {12}.{731}\;{\text{K}}_{{1}} - {697}.{75}\;{\text{K}}_{{2}} + {2}.{995}\;{\text{K}}_{{3}} + 0.{439}\;{\text{K}}^{{{2}/}}_{{1}} + {366}.{96}\;{\text{K}}^{{{2}/}}_{{2}} - {1}.0{66}\;{\text{K}}^{{{2}/}}_{{3}} + {2}.{235}\;{\text{K}}_{{1}} {\text{K}}_{{2}} + 0.{219}\;{\text{K}}_{{1}} {\text{K}}_{{3}} + {4}.{844}\;{\text{K}}_{{2}} {\text{K}}_{{3}}$$

6 $${\text{I}}\left( {{\text{R}}.{\text{S}}.\;{\text{mg}}/{\text{gds}}} \right) = {73}.{667} - {8}.{\text{372 K}}_{{1}} - {216}.{265}\;{\text{K}}_{{2}} + {3}0.{698}\;{\text{K}}_{{3}} + 0.{415}\;{\text{K}}^{{{2}/}}_{{1}} + {187}.{836}\;{\text{K}}^{{{2}/}}_{{2}} - {1}.{197}\;{\text{K}}^{{{2}/}}_{{3}} - 0.{3}0{4}\;{\text{K}}_{{1}} {\text{K}}_{{2}} - 0.0{21}\;{\text{K}}_{{1}} {\text{K}}_{{3}} - {19}.{31}0\;{\text{K}}_{{2}} {\text{K}}_{{3}}$$

7 $${\text{I}}\left( {{\text{T}}.{\text{P}}.\;{\text{mg}}/{\text{gds}}} \right) = - {1}.{3686} - 0.{1879}\;{\text{K}}_{{1}} + {2}.{879}\;{\text{K}}_{{2}} + 0.{4856}\;{\text{K}}_{{3}} + 0.0000{6}\;{\text{K}}^{{{2}/}}_{{1}} - {1}.0{177}\;{\text{K}}^{{{2}/}}_{{2}} - 0.0{293}\;{\text{K}}^{{{2}/}}_{{3}} + 0.{265}0\;{\text{K}}_{{1}} {\text{K}}_{{2}} - 0.00{86}\;{\text{K}}_{{1}} {\text{K}}_{{3}} - 0.00{62}\;{\text{K}}_{{2}} {\text{K}}_{{3}}$$

**Figure 4:**
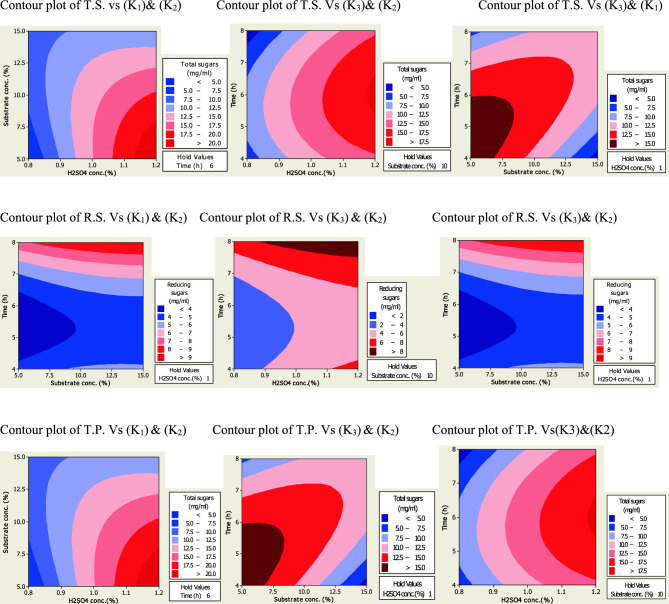
Contour plots of T.S., R.S. and T.P. (mg/gds) after chemical treatment of PPW.

**Figure 5:**
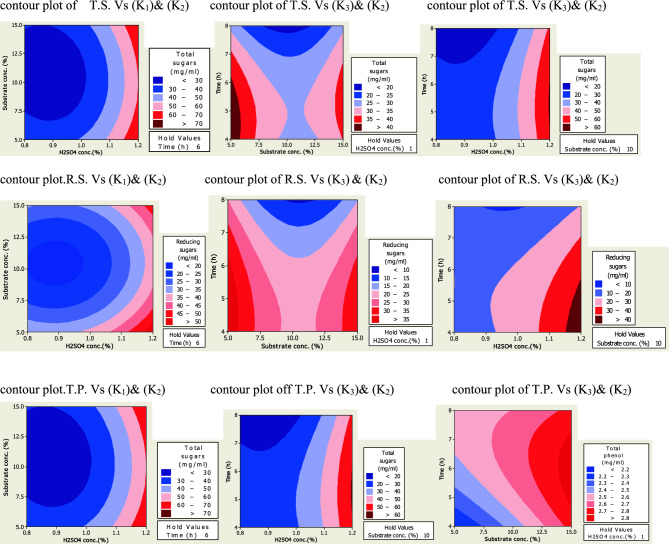
Contour plots of R.S., T.S., and T.P. (mg/gds) after thermo-chemical treatment of PPW.

The figures of 2D surface plots based on regression Eqs. (2–7) of T.S., R.S., and T.P. extracted during pretreatment at various conditions are depicted in Fig. 4 and 5. These plots describe the effect of various pretreatment conditions on the yield of fermentable sugars and phenolic compounds from treated PPW. The main colors are dark red, light pink and blue which represented highest, moderate and lowest yield, respectively. The acid assisted steam treatment caused more degradation of branched and complex substrate and eventually liberated more fermentable sugars.

In our study maximum amount of total sugar (T.S.) up to 720.00 mg/gds and reducing sugar (R.S.) up to 660.62 mg/gds were released after dilute sulphuric acid plus autoclaved treatment. Mushtaq et al.^52^ have reported high yield of total and reducing sugars upto 28.971and 7.163 mg/ml using alkali treated PPW. A previous report has described dilute sulphuric acid hydrolysis and sonication of PPW as a very promising process to release reducing sugars i.e., 57.03 g/kg^53^. It has been recorded that dilute acid treatment has significant effect on production of biogas with enhanced biomass reactivity and extraction of sugars^54^. In this study maximum amount of total phenolic compounds (T.P.C) obtained was 38.66 mg/gds after thermo-chemcial treatement employing 5% PPW with 0.8% loading of dilute sulphuric acid for 6 h.

Different studies have reported valuable extraction of phenolic compounds using using acidified solvent based mixtures. The acidified ethanolic extraction additionally avoids side reactions and product recovery is pure and stable. Many studies have investigated and compared acidic pretreatment with other advanced techniques and have revealed considerable effect on PPW degradation^21,51,55^. Their work has reported considerable extraction of sugars and other valuable bioactive components from PPW. Albeit in the present study no organic solvent was employed. Therefore, further work is required on these lines to increase purity as well as yields of acid treated PPW.

### FTIR spectroscopy

The FTIR test was conducted to assess the changes occurred at different stages in chemical and thermochemical treatments. The findings are presented in Fig. 2a–c.

The FTIR spectrum of raw PPW revealed a band between 3000 and 3500 cm^−1^, attributed to the presence of free and hydrogen bonded –OH group stretching vibrations derived from the polymers of cellulose, lignin and hemicellulose^56–58^. The peak at 1623 cm^−1^ may be ascribed to the existence of chemisorbed or physisorbed water molecules on the surface^59^. With reference to the peak at 1645.6 cm^−1^, it corresponded to the stretching vibrations of the C=C carbonyl group. Several bands between 1364 and 1103 cm^−1^ confirmed the lignin structure of substrate. Another peak at 1000 cm^−1^ attributed to the C–O–C elongations of the cellulose present in potato peel^60^.

In FTIR spectrum of acid treated substrate, the weak absorption peaks was observed at 2920 cm^−1^ reflected the stretching of C–H bond. The noticeable peak at 1010.1 cm^−1^ which resemble starch was attributed to C–O bond stretching^61^. In acid treated spectra, the strong peak at 1718.3 was attributed to carboxylic acid bond stretching. Additionally, two strong peaks observed at 1645 and 1623 cm^−1^ corresponding to C=O vibrations in the lignin part of PPW. The disappearance of both these peaks following acid treatment demonstrates that lignin part was degraded using sulphuric acid solution^62^. The displayed peaks at 1619 cm^−1^ and 1638 cm^−1^ indicated the presence of aromatic compounds in substrate particularly in lignin part confirming the bond cleavage^63^. Aryl OH group was detected at peak 1407 cm^−1^. The characteristic absorption peaks located at 1371 cm^−1^, 1381 cm^−1^ and 1010 cm^−1^ detected the stretching vibration of carbonyl (–CO) and methyl (–CH) moieties^64^. The region between 800 and 1500 cm^−1^ corresponding to cellulose, is a unique fingerprint region in which majority of bonds remained entangled. This indicates that regardless of acid treatment the cellulose maintains its chemical structure to the untreated one^65,66^. The C–H bond distortion in carbohydrate was quite visible in treated substrate as compared to the control substrate (Fig. 2b).

Spectrum of acid plus autoclaved substrate displayed narrow and lesser number of peaks confirming the degradation of substrate. The sharp peak displayed at 1617 cm^−1^ confirmed the C=C stretching vibrations of conjugated structures such as esters, ketone and quinone confirming the deconstruction of lignin part. Similar peaks at 1235 cm^−1^, 1313 cm^−1^, 1399 cm^−1^ showed moieties of –C–H and aromatic ring vibrations. Peaks at 1023 cm^−1^ and 1099 cm^−1^ depicted the stretching vibrations of C–O–C and C–O Bouhadjra et al.^64^. These absorption peaks confirmed the substantial deconstruction of PPW due to C–H, C=C, C–O–C and C–O vibrations proving the effectiveness of dilute acid as well as dilute acid assisted autoclaved pre-treatment techniques. The disaapearance of many peaks were observed in the region 800 and 1500 cm^−1^ as compared to untreated and acid treated spectras. This demostrates that cellullose part of PPW was decnstructed using acidn plus steaam treatment (Fig. 2c).

### Structural morphology

SEM microscopy was employed to reveal the topographical variations and deformations in substrate after pretreatments. The resulting SEM images of control and treated substrate are depicted in Fig. 3a, b.

Some pore and irregular crack were generated due to the acidic reaction that deform the structural bonds. Difference between structural arrangement of raw and treated samples was prominent and verified by electron micrographs. The raw sample exhibited a complex and compact order whereas treated samples generated a high degree of porosity (Fig. 3a, b). Same observations and disruptions have been described by Ben Taher et al.^55^. They have confirmed significant deformations, cracks and crevices formation in the micrographs of PPW after thermo-acidic pretreatments. In Fig. 3b, small pore like segments were observed after acid plus steam treatment. This indicates that the acid plus steam treatment may solubilize extracellular polymeric substance^67^. The ester bond was broken and many bounded phenolic acids were released. We can conclude that the combined treatment of acid plus steam produced smaller segments found in the micrographs. The structure of substrate was altered by the treatment and more bioactive components were released during this process.

Wang et al.^68^ have also reported perforated and disrupted substrate structure after ultrasonic assisted treatment using SEM micrographs. Frontuto et al.^69^ have also recorded disruption and deconstruction of PPW after using pulse electric field assisted extraction techniques. Sanusi et al.^70^ and Atitallah et al.^71^ have also revealed similar findings while evaluating the potential of PPW for bioethanol production.

### Degradation index of PPW

**Figure 6:**
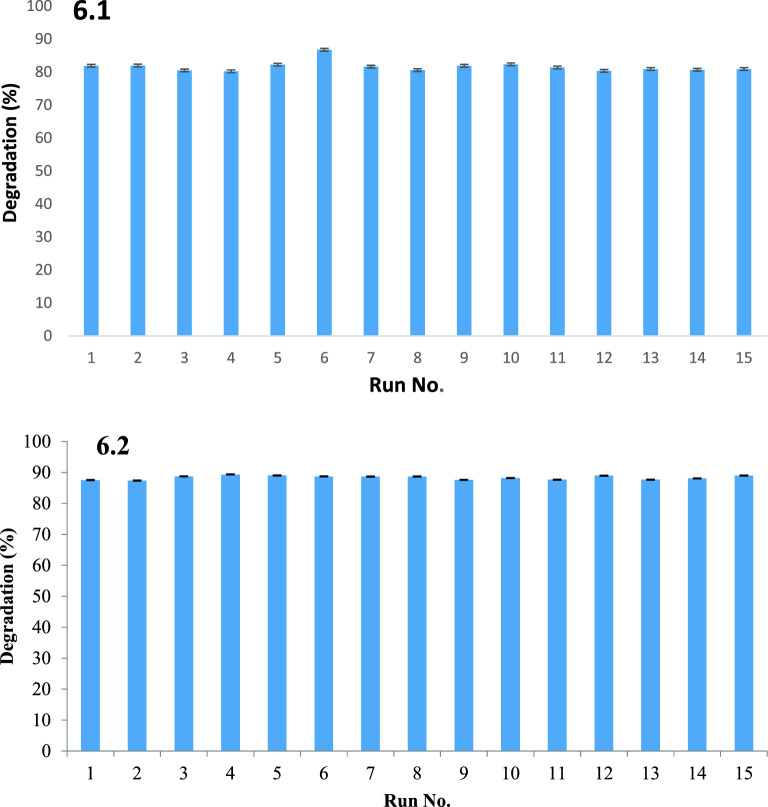
Degradation index of PPW; (**a**) acid treated, (**b**) acid plus steam treated.

Percentage degradation and mass balance of substrate was calculated was measured after completion of pretreatment process. Results revealed that in acidic treatment max. degradation of 86% was observed with loading of 1.2% acid and time period of 4 h as shown in Fig. 6a. In acid plus steam treatment, 89% of substrate degradation was recorded (Fig. 6b). A major improvement made in present study compared to expensive solvent assisted extraction is that the thermochemical extraction is the low cost, since sophisticated equipment as well as expensive organic solvents are not required. However, if required the value added organic bioactive compounds could be separated by employing specific solvents and advanced techniques.

### Acid saccharification of PPW

**Figure 7:**
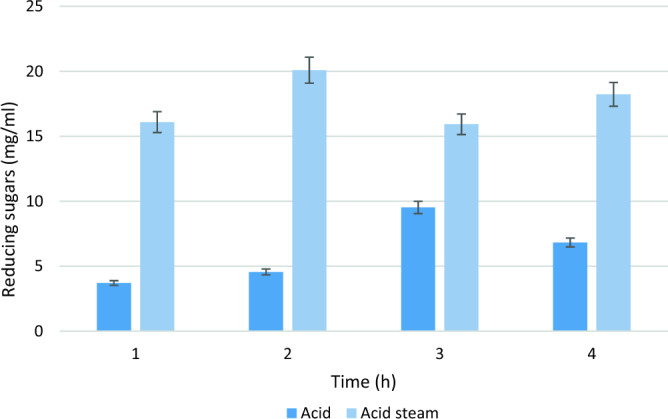
Extraction of reducing sugars at various time periods employing acid treated PPW.

Saccharification was calculated at different time intervals after acid and acid plus steam hydrolysis. Results revealed that maximum sugars were released after 6 h of acid assisted autoclaved treatment with saccharification level of 40%, whereas minimum saccharification of 31.8% was occurred after 10 h. In case of acid treated substrate, maximum saccharification of 19% was calculated as shown in Fig. 7. Interestingly, it has been recorded that different food wastes including mango, orange, banana and pineapple peels have been utilized for liberation of various valuable products such as fermentable sugars, dextran and hydroxymethylfurfural (HMF) production employing different methods^72–75^. Such biowastes can also be processed through the acid/acid assisted steam treatments reported in this study for rendering the achievements cost-effective.

## Conclusions

This study evaluated the effectiveness of dilute acid and acid assisted steam pre-treatment of potato peel waste. The extraction yields of total sugars and phenolic compounds was improved by combined acid and steam treatment. Statistical results of this study revealed maximum extraction of total and reducing sugars and phenolic compounds as 720.00, 660.62 and 38.66 mg/gds, respectively following the thermochemical treatment. The acid assisted autoclaved method proved to be very effective for the deconstruction of PPW and yielded the maximum release of both monomeric and oligomeric sugars. The cost-effective and simple strategy of dempolymerization of PPW into value added products is capable to provide various feedstocks to biotechnological processes with concomitant and sustainable management of solid food wastes.

## Data Availability

All the generated data is present in this manuscript.
